# Review of the genus *Metathrinca* Meyrick, 1908 (Lepidoptera, Xyloryctidae) from China, including descriptions of ten new species

**DOI:** 10.3897/zookeys.1287.186032

**Published:** 2026-08-07

**Authors:** Suyang Tang, Houhun Li

**Affiliations:** 1 College of Life and Geographic Sciences, Key Laboratory of Biological Resources and Ecology of Pamirs Plateau in Xinjiang, Kashi University, Kashi 844000, China College of Life Sciences, Nankai University Tianjin China; 2 College of Life Sciences, Nankai University, Tianjin 300071, China College of Life and Geographic Sciences, Key Laboratory of Biological Resources and Ecology of Pamirs Plateau in Xinjiang, Kashi University Kashi China

**Keywords:** Microlepidoptera, Gelechioidea, new species, taxonomy

## Abstract

Eighteen species of the genus *Metathrinca* Meyrick, 1908 in China are reviewed. Among them, ten species are described as new: *M. breviprocessa* Li, **sp. nov**., *M. crassiprocessa* Li, **sp. nov**., *M. digitiloba* Li, **sp. nov**., *M. fortispina* Li, **sp. nov**., *M. graciliphalla* Li, **sp. nov**., *M. licina* Li, **sp. nov**., *M. lingugnathos* Li, **sp. nov**., *M. nigrivenosa* Li, **sp. nov**., *M. punctuatella* Li, **sp. nov**., and *M. sublunata* Li, **sp. nov**. In addition, the females of *M. meihuashana* Wang, Zheng & Li, 2000 and *M. intacta* (Meyrick, 1938) are reported for the first time. Images of adults and illustrations of genitalia are provided, except those of the three species published in 2025. A key to all the known *Metathrinca* species in China is included.

## Introduction

The genus *Metathrinca* was initially defined and placed in the family Xyloryctidae by Meyrick (1908), with *M. ancistrias* (Meyrick, 1906) designated as the type species. *Metathrinca* represents a typical oriental group of insects and currently comprises 27 known species (Zuo et al. 2025; Sterling et al. 2025, 2026).

Prior to this study, eight *Metathrinca* species had been recorded in China (Zuo et al. 2025). The aim of the present study is to review the *Metathrinca* species in China, to describe ten new species, and to report the females of two known species for the first time based on the Chinese material.

## Materials and methods

Specimens used in this study were collected by light traps. Genitalia slides were prepared using the methods introduced by Li (2002). Images of adults and genitalia were taken with a Leica M205A microscope, coupled with the Leica Application Suite X software.

All the studied specimens, including the types, are deposited in the Insect Collection of Tianjin Natural History Museum, Tianjin, China (**TJNHM**).

### Abbreviations

**leg**. legit (Latin);

**NHMUK** Natural History Museum, Department of Zoology, London, UK;

**NKU** Insect Collection, College of Life Sciences, Nankai University, Tianjin, China;

**TD** Type depository;

**TJNHM** Insect Collection of Tianjin Natural History Museum, Tianjin, China;

**TL** Type locality.

## Taxonomic account

### Metathrinca

Taxon classificationAnimaliaLepidopteraXyloryctidae

Meyrick, 1908

5C92C223-7750-5B50-937F-1ECFDEF0BC89

Metathrinca Meyrick, 1908: 625. Type species: Ptochoryctis ancistrias Meyrick, 1906, by original designation.

#### Diagnosis.

**Figure 1.**
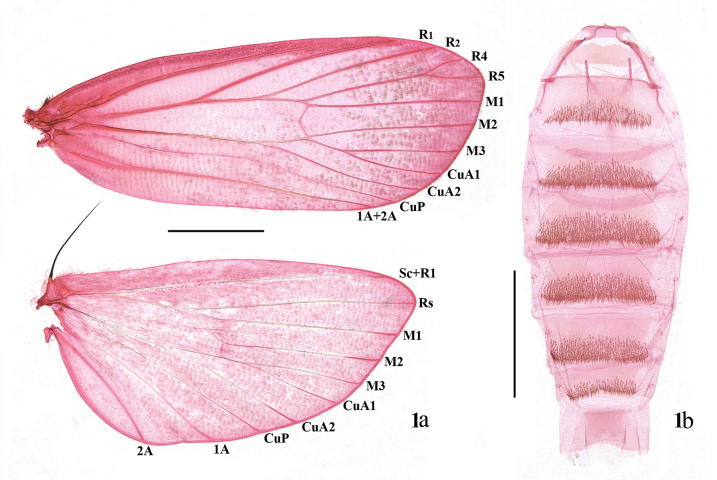
Wing venation and abdomen. *Metathrinca lingugnathos* Li, sp. nov., slide no. TSY24521. **1a**. Forewing and hindwing; **1b**. Abdomen. Scale bars: 2.0 mm.

The genus *Metathrinca* is diagnosed by the combinations of the following features: the head with spreading lateral tufts, the labial palpi curved upwards, the antennae bipectinate in male and filiform in female, and the forewings typically white with an outwardly arched subterminal line and sometimes also with a terminal line; R_3_ absent, R_4_ and R_5_ stalked in the forewing, and M_3_ and CuA_1_ stalked in the hindwing (Fig. 1a), and the abdominal tergites possessing a band of spines posteriorly (Fig. 1b). The male genitalia are distinguished by the gnathos with dense protuberances, the developed saccular process usually bearing setae and a row or a cluster of spines, and the simple phallus rarely with a cornutus. The female genitalia are distinguished by the small subcircular or elongate ostium bursae, the ductus bursae mostly longer than the elliptical or ovoid corpus bursae, and the signum, if present, usually with a sclerotized transverse band or a ridge.

##### Key to *Metathrinca* species in China

**Table:** 

1	Forewing with markings	**2**
–	Forewing without markings except subterminal and terminal lines	**5**
2	Forewing with pale brown spot at end of cell (Fig. 2)	***M. punctuatella* sp. nov**.
–	Forewing without spot at end of cell	**3**
3	Gnathos separated into two lobes or bilobed distally	**4**
–	Gnathos not divided (Fig. 28)	***M. nigrivenosa* sp. nov**.
4	Gnathos separated into two lobes, saccular process exceeding costal margin of valva (Fig. 31)	***M. tsugensis***
–	Gnathos bilobed distally, saccular process not exceeding costal margin of valva (Wang et al. 2000: fig. 2)	***M. fopingensis***
5	Uncus pointed at apex	**6**
–	Uncus concave at apex	**8**
6	Phallus with a spine-shaped preapical process (Fig. 22)	***M. fortispina* sp. nov**.
–	Phallus without a preapical process	**7**
7	Phallus curved, valva with a subtriangular process beyond base of saccular process (Fig. 19)	***M. crassiprocessa* sp. nov**.
–	Phallus straight, valva without a subtriangular process (Fig. 15)	***M. sublunata* sp. nov**.
8	Saccular process with one or two additional apical spine(s)	**9**
–	Saccular process with a cluster of spines only	**12**
9	Saccular process with two apical spines (Zuo et al. 2025: fig. 1)	***M. bispina***
–	Saccular process with one apical spine	**10**
10	Saccular process curved in L shape, longer than 1/2 length of valva (Fig. 26)	***M. lingugnathos* sp. nov**.
–	Saccular process curved in C shape, shorter than 1/2 length of valva	**11**
11	Gnathos with ventral plate subrectangular, valva narrowed from base to rounded apex (Zuo et al. 2025: fig. 3)	***M. zhengi***
–	Gnathos with ventral plate subtrapezoidal, valva wide and parallel sided basally, slightly narrowed to obtuse apex distally (Zuo et al. 2025: fig. 2)	***M. mutativalvis***
12	Gnathos broadly subcircular, not bilobed (Wang et al. 2000: fig. 5)	***M. meihuashana***
–	Gnathos bilobed	**13**
13	Phallus serrate on one side distally, valva almost truncate at apex (Wang et al. 2000: fig. 1)	***M. argentea***
–	Phallus not serrated distally, valva pointed or rounded at apex	**14**
14	Valva triangularly produced to a pointed apex distally	**15**
–	Valva narrowed to a rounded apex distally or produced to a papillary process apically	**17**
15	Phallus longer than valva (Fig. 23)	***M. graciliphalla* sp. nov**.
–	Phallus shorter than valva	**16**
16	Saccular process with densely clustered spines of equal length running from middle to apex, apically not exceeding costal margin of valva (Fig. 18)	***M. breviprocessa* sp. nov**.
–	Saccular process with fine spines medially and with stronger sparse spines distally, apically exceeding costal margin of valva (Fig. 20)	***M. digitiloba* sp. nov**.
17	Valva narrowed to a rounded apex, saccular process extending upward to below costal margin of valva, phallus slightly dilated distally (Fig. 24)	***M. intacta***
–	Valva produced to a papillary process apically, saccular process extending upward far beyond costal margin of valva, phallus tapered distally (Fig. 25)	***M. licina* sp. nov**.

### Metathrinca argentea

Taxon classificationAnimaliaLepidopteraXyloryctidae

Wang, Zheng & Li, 2000

53A72E30-C6B1-5438-81E6-6E14CFF890AF

Figs 2, 17, 32

Metathrinca argentea Wang, Zheng & Li, 2000: 230. TL: China (Henan: Mt. Jigong, Xinyang). TD: NKU.

#### Material examined.

***Holotype***: China: • ♂; Henan Province, Xinyang, Mt. Jigong; 700 m; 13 July 1997; leg. HH Li. ***Paratypes***: China: • 1♂1♀; same data as holotype; • 1♂; Fujian, Mt. Meihuashan; 21 July 1988 • 1♂2♀; Anhui, Yuexi County, Wenquan Town; 5 Aug. 1995; leg. XF Hu • 1♂13♀; Sichuan, Luxian County; 26 July 1995; leg. YX Zeng.

**Figures 2–9.**
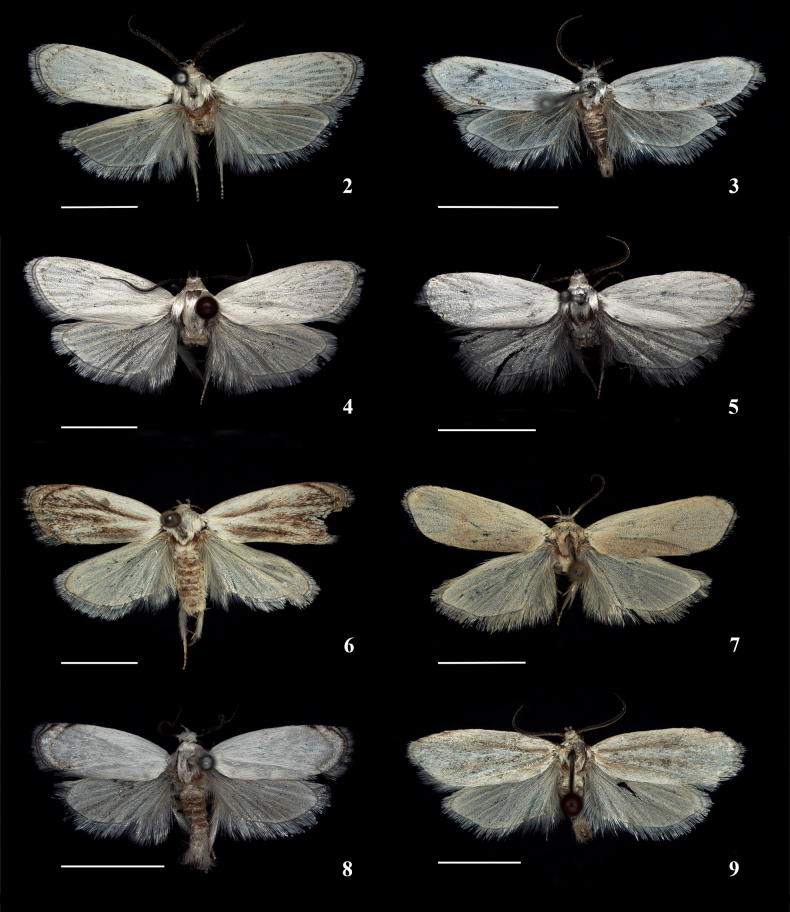
Adults of *Metathrinca* spp. **2**. *M. argentea* Wang, Zheng & Li, 2000; **3**. *M. breviprocessa* Li, sp. nov., ♂, holotype; **4**. *M. crassiprocessa* Li, sp. nov., ♂, holotype; **5**. *M. digitiloba* Li, sp. nov., ♂, holotype; **6**. *M. fopingensis* Wang, Zheng & Li, 2000; **7**. *M. fortispina* Li, sp. nov., ♂, holotype; **8**. *M. graciliphalla* Li, sp. nov., ♂, holotype; **9**. *M. intacta* (Meyrick, 1938). Scale bars: 5.0 mm.

**Figures 10–16.**
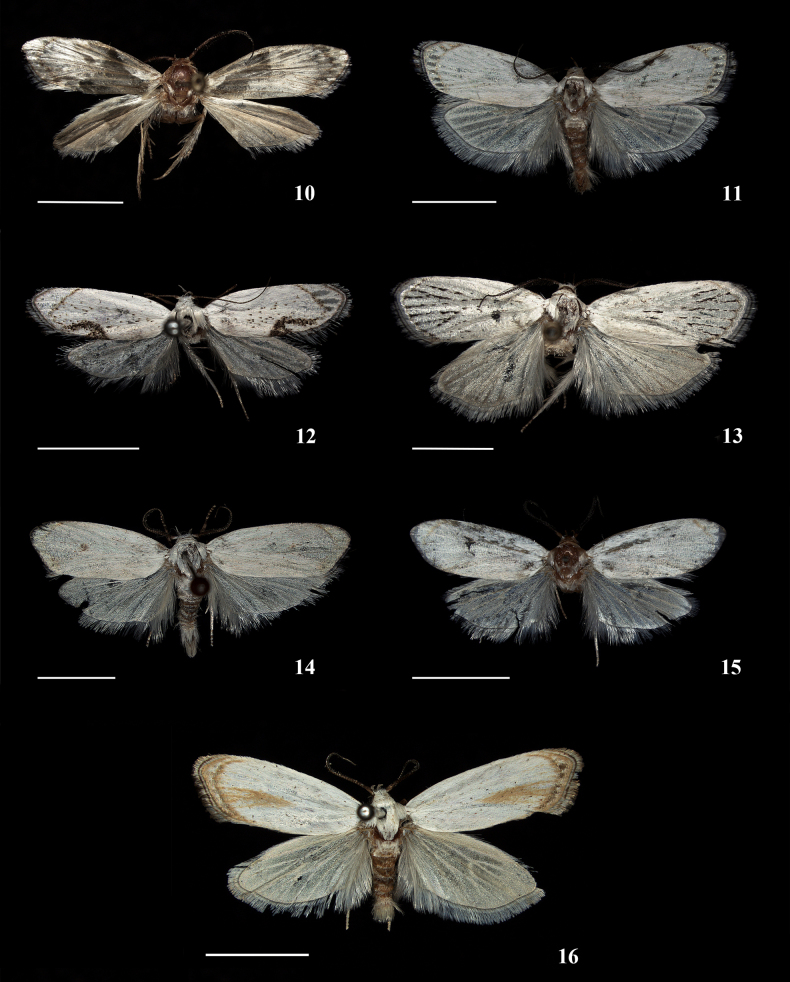
Adults of *Metathrinca* spp. **10**. *M. licina* Li, sp. nov., ♂, holotype; **11**. *M. lingugnathos* Li, sp. nov., ♂, holotype; **12**. *M. meihuashana* Wang, Zheng & Li, 2000; **13**. *M. nigrivenosa* Li, sp. nov., ♂, holotype; **14**. *M. punctuatella* Li, sp. nov., ♂, holotype; **15**. *M. sublunata* Li, sp. nov., ♂, holotype; **16**. *M. tsugensis* (Kearfott, 1910). Scale bars: 5.0 mm.

**Figures 17–24.**
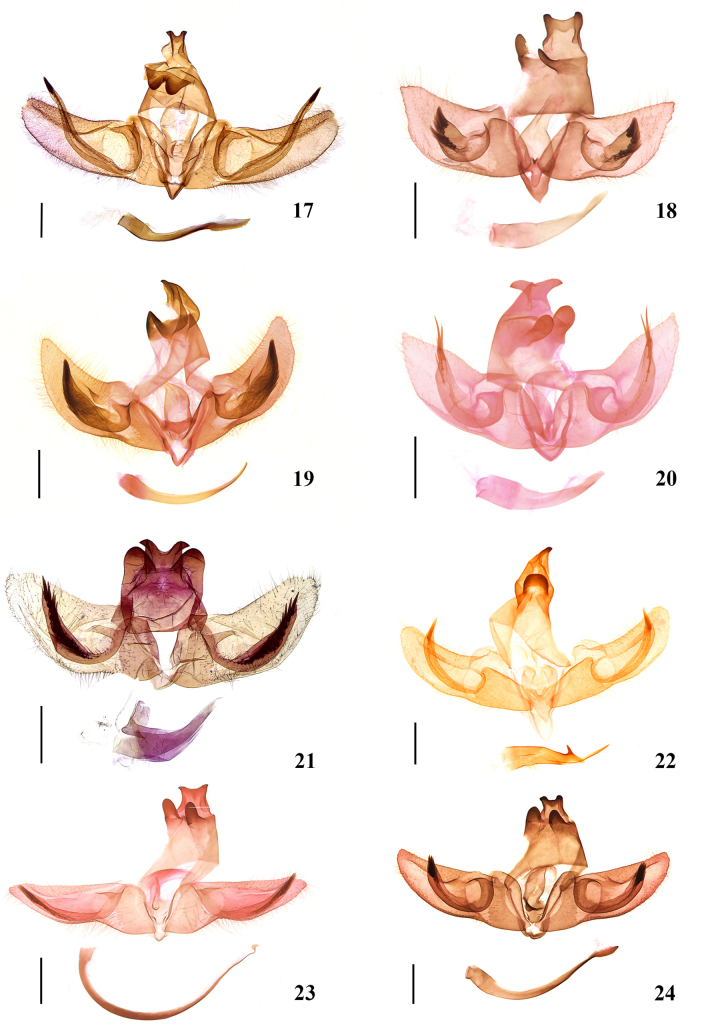
Male genitalia of *Metathrinca* spp. **17**. *M. argentea* Wang, Zheng & Li, 2000, slide no. L97379, holotype; **18**. *M. breviprocessa* Li, sp. nov., slide no. TSY25423, holotype; **19**. *M. crassiprocessa* Li, sp. nov., slide no. TSY24582, holotype; **20**. *M. digitiloba* Li, sp. nov., slide no. TSY25262, holotype; **21**. *M. fopingensis* Wang, Zheng & Li, 2000, slide no. W95184, holotype; **22**. *M. fortispina* Li, sp. nov., slide no. TSY25073, holotype; **23**. *M. graciliphalla* Li, sp. nov., slide no. TSY25420, holotype; **24**. *M. intacta* (Meyrick, 1938), slide no. TSY25418. Scale bars: 0.5 mm.

**Figures 25–31.**
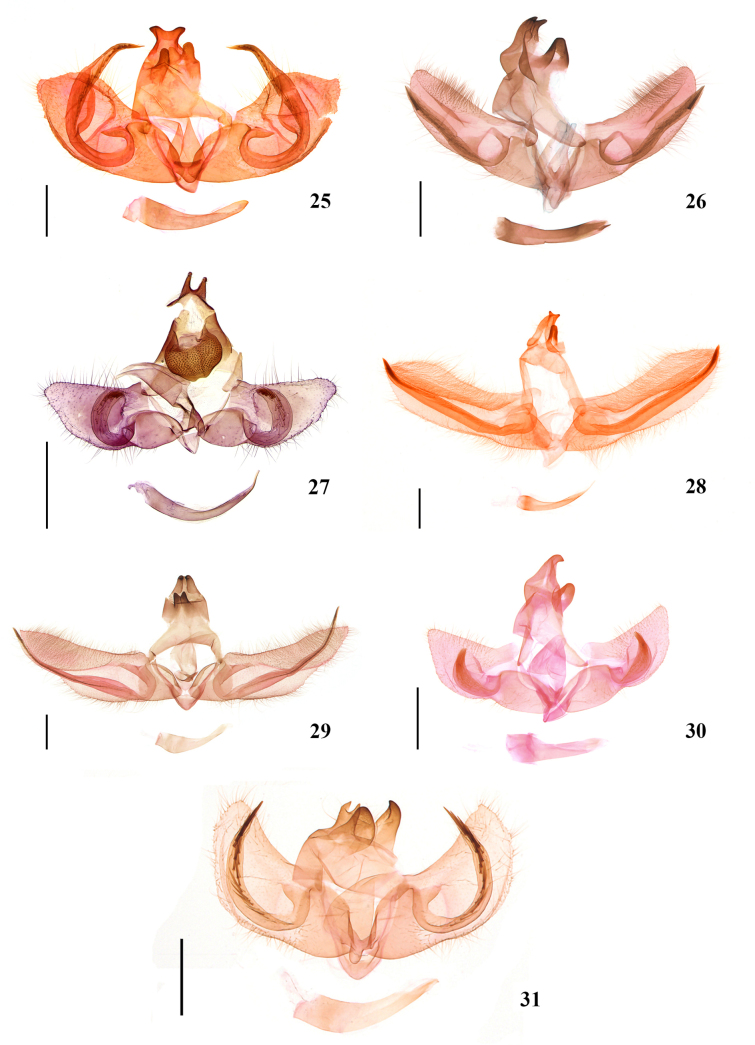
Male genitalia of *Metathrinca* spp. **25**. *M. licina* Li, sp. nov., slide no. TSY24504, holotype; **26**. *M. lingugnathos* Li, sp. nov., slide no. TSY25419, holotype; **27**. *M. meihuashana* Wang, Zheng & Li, 2000, slide no. L93025, holotype; **28**. *M. nigrivenosa* Li, sp. nov., slide no. TSY24488, holotype; **29**. *M. punctuatella* Li, sp. nov., slide no. TSY25421, holotype; **30**. *M. sublunata* Li, sp. nov., slide no. TSY25277, holotype; **31**. *M. tsugensis* (Kearfott, 1910), slide no. TSY25184. Scale bars: 0.5 mm.

**Figures 32–37.**
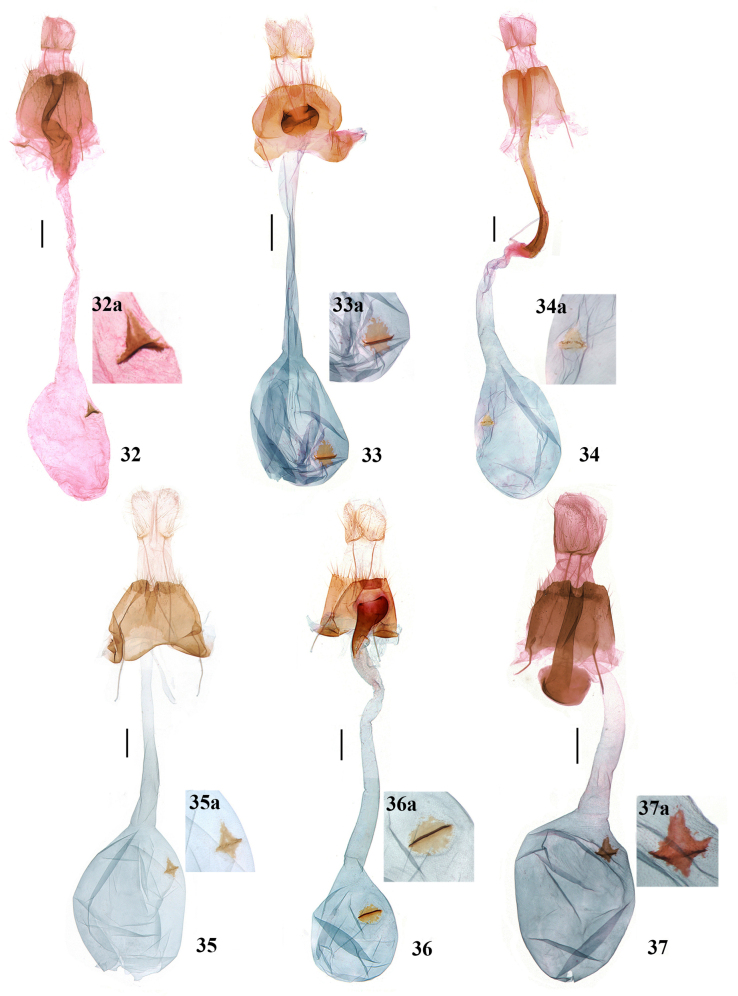
Female genitalia of *Metathrinca* spp. **32**. *M. argentea* Wang, Zheng & Li, 2000, slide no. TSY24419; **33**. *M. breviprocessa* Li, sp. nov., slide no. TSY25404, paratype; **34**. *M. crassiprocessa* Li, sp. nov., slide no. TSY25402, paratype; **35**. *M. fopingensis* Wang, Zheng & Li, 2000, slide no. TSY24540; **36**. *M. fortispina* Li, sp. nov., slide no. TSY25407; **37**. *M. intacta* (Meyrick, 1938), slide no. TSY25416. **32a–37a**. Enlarged view of signum. Scale bars: 0.5 mm.

#### Additional material examined.

China: • 1♂3♀; Chongqing, Mt. Jinyun; 29–30 July 2010; leg. XC Du & CW Bi • 1♂; Fujian, Hua’an County, Mt. Gongya; 24.93°N, 107.40°E; 1022 m; 29 July 2020; leg. MJ Qi & XY Jin; slide no. TSY24369♂ • 1♂; Guangdong, Shaoguan, Shimentai Reserve, Huawu Village; 24.72°N, 113.07°E; 403 m; 28 May 2021; leg. XH Zuo • 1♂; Guangdong, Shaoguan, Chebaling Reserve, Piaoliugu; 24.72°N, 114.26°E; 360 m; 31 May 2021; leg. XH Zuo; slide no. TSY24307 • 1♂; Guangxi, Hechi City, Huanjiang County, Yangmei’ao; 1180 m; 23 July 2015; leg. MQ Yang & G-E Lee; slide no. TSY24493 • 4♂; Guizhou, Jiangkou County, Taiping Town; 27.84°N, 108.77°E; 528 m; 13–16 July 2019; leg. MR Xing, BX Zhao & H Sun; slide nos. TSY24222, TSY24256, TSY24292, TSY25167 • 2♂2♀; Hainan, Ledong County, Jianfengling Nature Reserve; 18.44°N, 108.52°E; 770 m; 30 May, 3–5 June 2015; leg. PX Cong, W Guan & S Hu; slide nos. TSY24264♂, TSY24351♂, TSY24419♀ • 1♂; Henan, Luoshan County, Lingshan Temple; 350 m; 22 May 2000; leg. HL Yu; slide no. TSY24513 • 3♂10♀; Hubei, Yingshan County, Taohuachong; 30.99°N, 116.03°E; 635 m; 23–28 June 2014; leg. W Guan & MQ Yang; • 1♀; Hunan, Yizhang County, Mangshan Reserve, Xiangsikeng; 24.95°N, 112.98°E; 1326 m; 25 July 2020; leg. H Sun, XH Zuo & ZL Tao; slide no. TSY24450 • 1♂; Hunan, Zhangjiajie; 10 Aug. 2001; 650 m; leg. HH Li & XP Wang; slide no. TSY24490 • 1♂; Jiangxi, Mt. Jiulian; 21 July 2006; leg. JS Xu & WC Li; slide no. TSY25120 • 3♂; Sichuan, Leshan, Mt. E’mei; 29.57°N, 103.40°E; 790 m; 19–23 July 2021; slide nos. TSY24531, TSY24533, TSY24536 • 2♂1♀; Taiwan, Taipei, Yilan County, Mt. Fu; 420 m; 31 July–1 Aug. 2006; leg. HH Li & XC Du; slide no. TSY25192♂ • 3♂; Zhejiang, Mt. Tianmu, Kaishandian; 1140 m; 17 Aug. 1999; leg. HH Li et al.; slide nos. TSY24547, TSY24548.

#### Diagnosis.

Adult (Fig. 2). Wingspan 18.0–28.0 mm. *Metathrinca argentea* is similar to *M. intacta* in the male genitalia. It can be distinguished by the saccular process exceeding the apex of the valva, the valva obtuse at the apex, and the phallus with distal 1/3 inflated (Fig. 17); in *M. intacta*, the valva is bluntly rounded at the apex, the saccular process does not extend to the apex of the valva, and the distal 1/6 of the phallus is slightly inflated (Clarke 1955: 446, pl. 222, figs 4–4b). *Metathrinca argentea* can be distinguished from *M. intacta* in the female genitalia by the antrum subrectangularly widened anteriorly and the signum is subtriangularly shaped (Fig. 32); in *M. intacta*, the antrum is roundly dilated anteriorly and the signum is more or less cross-shaped (Fig. 37).

#### Distribution.

China (Anhui, Chongqing, Fujian, Guangdong, Guangxi, Guizhou, Hainan, Henan, Hubei, Hunan, Jiangxi, Sichuan, Taiwan, Zhejiang).

### Metathrinca bispina

Taxon classificationAnimaliaLepidopteraXyloryctidae

Li, 2025

FBFD9CD7-65B5-5FF1-8CF4-3BB7B91480C9

Metathrinca bispina Zuo, Tang & Li, 2025: 152. TL: China (Sichuan: Bifeng Gorge, Ya’an). TD: NKU.

#### Type materials and descriptions.

See Zuo et al. (2025).

#### Distribution.

China (Anhui, Fujian, Gansu, Guangxi, Guizhou, Hainan, Hubei, Hunan, Jiangxi, Sichuan, Yunnan, Zhejiang).

### Metathrinca breviprocessa

Taxon classificationAnimaliaLepidopteraXyloryctidae

Li sp. nov.

5A2E4BB0-E6D5-57DF-9FEA-65E8F5E91517

https://zoobank.org/371C5EEF-D0C1-4C57-A109-0159331C9FBA

Figs 3, 18, 33

#### Type material.

***Holotype***: China: • ♂; Yunnan Province, Pu’er City, Taiyanghe Nature Reserve; 22.68°N, 101.03°E; 1450 m; 13 Aug. 2016; leg. KJ Teng, J-E Li & T Wang; slide no. TSY25423. ***Paratypes***: China: • 27♂1♀; same data as holotype; slide nos. TSY24500♂, TSY24505♂, TSY24557♂, TSY25404♀ • 1♂; Yunnan, Jingdong County, Back Hill of Lac Research Institute; 1140 m; 20 July 2013; leg. ZG Zhang; slide no. TSY25271.

#### Diagnosis.

The new species is characterized in the male genitalia by the strongly divided gnathos separated from the base, the broad subparallelogram-shaped valva, and the sacculus having a short saccular process bearing dense fine spines of the same length. The female genitalia can be distinguished from those of its congeners by having a transversely elliptical antrum with triangular plates posteriorly.

#### Description.

Adult (Fig. 3). Wingspan 15.5–20.0 mm.

***Head***. Vertex and frons white. Labial palpus curved upward, not reaching level of vertex; white, segment I pale brown on outer side, segment II pale brown in basal 1/2 on outer side. Male antenna with scape brown, flagellum dark brown in basal 1/3, yellow ringed with pale brown in distal 2/3; female antenna brown.

***Thorax***. Mesonotum, tegula and forewing white. Forewing narrow at base, subparallel from near base to before basal 2/3, slightly narrowed from distal 1/3 to obtuse apex, termen oblique; subterminal line pale yellowish brown, dark brown near tornus; terminal line yellow; fringe white, tipped with pale brown, with a brownish yellow line near middle. Hindwing and fringe white, tinged with yellow. Foreleg brown; midleg white, tarsus pale brown; hindleg white, tarsus pale brown in distal 1/3.

***Abdomen***. Male genitalia (Fig. 18). Uncus subquadrate, heavily sclerotized laterally and distally; apex broadly and shallowly concave medially, forming paired lateral lobes obliquely extending outward. Gnathos separated from base, forming two thumb-shaped lobes with dense protuberances. Tegumen subtrapezoidal, shortly extended anterolaterally. Valva broad, subparallelogram-shaped, subparallel from base to before triangularly produced apex; costal margin slightly concave near base; ventral margin shallowly concave beyond basal 1/3. Sacculus slightly narrowed to below costal margin, < 1/4 length of ventral margin of valva; saccular process extended outward as a short rectangular band at base, curved in C shape, open to costal margin, widened and arched ventrad to ventral margin of valva medially, narrowing towards below costal margin, acute at apex, distal 1/2 bearing a cluster of dense strong spines of equal length. Juxta narrowed from subbase to apex, bilobed distally. Saccus triangular, broad basally, slightly narrowed to apex. Phallus almost equal to valva in length, slightly curved, tapered to apex.

Female genitalia (Fig. 33, 33a). Papilla anales subovate, with short setae. Apophyses posteriores ~2× length of apophyses anteriores. Eighth abdominal segment with short setae on posterior margin. Antrum strongly sclerotized, transversely elliptical, with paired small sclerotized, triangular plates posteriorly. Ductus bursae ~2× length of corpus bursae. Corpus bursae elliptical; signum rhomboidal, with a sclerotized ridge in middle (Fig. 33a).

#### Distribution.

China (Yunnan).

#### Etymology.

The specific epithet is derived from the Latin *brevi*- and *processus*, referring to the relatively short saccular process of the male genitalia.

### Metathrinca crassiprocessa

Taxon classificationAnimaliaLepidopteraXyloryctidae

Li sp. nov.

9F28513F-2260-534D-BCDD-E452749E83F5

https://zoobank.org/154C4262-1BA5-48ED-A1B7-640ABD7EBF0C

Figs 4, 19, 34

#### Type material.

***Holotype***: China: • ♂; Hainan Province, Qiongzhong County, Limushan Reserve Core Zone; 19.17°N, 109.74°E; 600–700 m; 3 May 2014; leg. TT Liu, W Guan & XM Hu; slide no. TSY24582. ***Paratypes***: China: • 11♂1♀; same data as holotype, except dated 2–5 May 2014; slide nos. BX15036♂, BX15038♂, BX15040♂, TSY24528♂, TSY24564♂, TSY24591♂, TSY24613♂, TSY25241♂, TSY25243♂, TSY25402♀, TSY25424♂ • 1♂; Hainan, Baisha County, Yuanmen Township; 19.08°N, 109.52°E; 14 April 2014; leg. TT Liu, W Guan & XM Hu; slide no. BX15034 • 1♂; Hainan, Ledong County, Tianchi, Jianfengling; 18.74°N, 108.84°E; 1050 m; 30 April 2013; leg. YH Sun, W Guan & TT Liu; slide no. BX15004 • 1♂; Hainan, Ledong County, Jianfengling Nature Reserve; 18.44°N, 108.52°E; 770 m; 28 May 2015; leg. PX Cong, W Guan & S Hu; slide no. TSY24402 • 1♂; Hainan, Tianchi, Jianfengling; 18.73°N, 108.87°E; 787 m; leg. QY Wang, SR Li & MT Chen; slide no. TSY25341 • 1♂; same locality as above; leg. X Bai, SN Qian & WD Qi; slide no. TSY25403 • 1♂; Hainan, Bawangling, East-2 Workstation; 1000 m; 23 April 2009; leg. BB Hu & Q Jin; slide no. WQY15136 • 1♂; Chongqing, Tudiyan, Mt. Simian; 1200 m; 16 July 2012; leg. YH Sun & AH Yin; slide no. TSY24523 • 1♂; Sichuan, Leshan City, Mt. Emei; 29.57°N, 103.40°E; 790 m; 19 July 2021; leg. S Yu, XJ Zhu & D Zhang; slide no. TSY24242 • 1♂; same locality as above; 847 m; 22 July 2021; slide no. TSY24180 • 1♂; Yunnan, Taiyanghe Nature Reserve; 1450 m; 16 May 2014; leg. ZG Zhang; slide no. TSY25309.

#### Diagnosis.

The new species is characterized in the male genitalia by the beak-like uncus in a lateral view and the valva with a subtriangular process situated beyond the base of the saccular process. The female genitalia are distinguished by the slender ductus bursae sclerotized in the basal 1/2 and membranous in the distal 1/2.

#### Description.

Adult (Fig. 4). Wingspan 18.5–24.0 mm.

***Head***. Vertex and frons white. Labial palpus curved upward, exceeding beyond vertex; brown, in male segment II white in basal 1/3 on dorsal surface, in female white on inner surface. Antenna with scape dark brown, flagellum black.

***Thorax***. Mesonotum, tegula and forewing white. Forewing narrow at base, gradually widened to beyond distal 1/3, thereafter narrowed to rounded apex, termen obliquely obtuse; white, tinged with pale yellow; subterminal line yellow; fringe white, with a dark brown subbasal line. Hindwing and fringe white. Foreleg with femur white, tibia and tarsus dark brown, tarsomeres yellowish brown at apices; midleg white, tarsus yellowish brown; hindleg white, distal four tarsomeres pale brown, with white apices.

***Abdomen***. Male genitalia (Fig. 19). Uncus broad at base, tapered to pointed apex, beak-like distally in lateral view. Gnathos wide at base, narrowed to apex, with dense protuberances. Tegumen subtrapezoidal posteriorly, extending to rounded anterior end laterally. Valva parallel sided basally, narrowed to rounded apex distally; costal margin concave near base; ventral margin concave at basal 1/3. Sacculus narrow, nearly band-shaped, extending close to costal margin of valva, < 1/4 length of ventral margin of valva ventrally; saccular process extended outward as a short bar at base, curved in U shape and smooth basally, widened medially, narrowed from distal 1/3 to pointed apex, densely covered with fine spines from near base to apex, becoming denser towards apex; small subtriangular process placed beyond base of saccular process, extending outward, wide at base, narrowed to pointed apex. Juxta narrowed from near base to apex, distal 1/4 bilobed. Saccus triangular. Phallus slender, tapering from base to apex, slightly shorter than valva, curved.

Female genitalia (Fig. 34, 34a). Papilla anales subquadrate, sparsely setose. Apophyses posteriores ~2× length of apophyses anteriores. Eighth sternum emarginated medially on posterior margin. Ostium bursae subcircular, positioned at posterior margin of eighth sternum. Ductus bursae elongate, ~ 2.5× length of corpus bursae, basal 1/2 sclerotized, slender, distal 1/2 membranous, broadened toward corpus bursae; ductus seminalis arising from posterior 1/2 of ductus bursae. Corpus bursae elliptical; signum subtriangular, with a weakly sclerotized transverse ridge in middle (Fig. 34a).

#### Distribution.

China (Chongqing, Hainan, Sichuan, Yunnan).

#### Etymology.

The specific epithet is derived from the Latin *crassi*- and *processus*, referring to the wide saccular process of the valva in the male genitalia of this species.

### Metathrinca digitiloba

Taxon classificationAnimaliaLepidopteraXyloryctidae

Li sp. nov.

E999EAA0-D6D2-54E8-93DA-2043CE1C85EF

https://zoobank.org/5F0368FD-155D-4CF7-A579-CFC9D6ABB7B5

Figs 5, 20

#### Type material.

***Holotype***: China: • ♂; Hainan Province, Diaoluoshan National Nature Reserve; 18.43°N, 109.52°E; 922 m; 26 May 2015; leg. PX Cong, W Guan & S Hu; slide no. TSY25262. ***Paratypes***: China: • 4♂; same data as holotype, except dated 26–27 May 2015; slide nos. TSY24386, TSY25273 • 12♂; Hainan, Ledong County, Jianfengling Nature Reserve; 18.74°N, 108.87°E; 770 m; 13–16 July 2014; leg. PX Cong, W Guan & S Hu; slide nos. BX15050, TSY25288, TSY25305, TSY25306, WQY15120, WQY15125, WQY15127, WQY15137 • 3♂; Hainan, Ledong County, Jianfengling Nature Reserve; 18.44°N, 108.52°E; 770 m; 30 May–4 June 2015; leg. PX Cong, W Guan & S Hu; slide nos. TSY25284, TSY25298.

#### Diagnosis.

The new species is similar to *M. intacta* (Meyrick, 1938) in the male genitalia. It can be separated from the latter by the valva triangularly produced from distal 1/4 to a pointed apex, the gnathos without a small process between the two lobes, and the juxta bilobed distally; in *M. intacta*, the valva is slightly narrowed from distal ~1/3 to a rounded apex, the gnathos possesses a small process between the two lobes, and the juxta is not bilobed distally (Fig. 24).

#### Description.

Adult (Fig. 5). Wingspan 18.0–21.5 mm.

***Head***. Vertex and frons snowy white. Labial palpus curved upward to vertex; white, except segments I and II yellowish brown on outer side. Male antenna with scape yellowish brown; flagellum basally with a few flagellomeres dark yellowish brown, remaining flagellomeres yellow ringed with brown.

***Thorax***. Mesonotum, tegula and forewing white. Forewing narrow at base, gradually widened to beyond basal 2/3, then slightly narrowed to obtusely rounded apex, termen obliquely obtuse; subterminal line pale yellow; fringe white, with a yellow subbasal line, distally grey, sparsely tipped with black. Hindwing and fringe white, tinged with yellow. Foreleg with femur brown, tibia and tarsus dark brown, distal tarsomeres dark yellowish brown; midleg white, tarsus brown intermixed with white; hindleg white, distal four tarsomeres brown, ringed with white at apices.

***Abdomen***. Male genitalia (Fig. 20). Uncus wide at base, apex concave in broad V shape, forming a pair of horn-like lobes spreading laterally; lateral margin concave below lateral lobes. Gnathos bifid, forming two broadened, thumb-shaped lobes. Tegumen subtrapezoidal, not distinctly extended anterolaterally. Valva broad at base, slightly narrowed to basal 3/4, distal 1/4 triangularly produced to a pointed apex; costal margin concave medially; ventral margin slightly concave at basal 1/4. Sacculus extending close to costal margin of valva, reaching 1/4 length of ventral margin of valva ventrally; saccular process triangularly extended outward at base, curved basally, forming a U shape open to costal margin, medially widened and arched to above ventral margin of valva, narrowing and oblique dorsad to apex, with a line of fine spines medially, with a cluster of stronger sparse spines distally, apically exceeding costal margin of valva. Juxta broad near base, narrowed and bilobed distally. Saccus subtriangular. Phallus ~ 6/7 length of valva, broad at base, tapered towards apex, slightly curved.

Female unknown.

#### Distribution.

China (Hainan).

#### Etymology.

The specific epithet is derived from the Latin *digitus* and *lobus*, referring to the bilobed, digitate gnathos of the male genitalia.

### Metathrinca fopingensis

Taxon classificationAnimaliaLepidopteraXyloryctidae

Wang, Zheng & Li, 2000

9811B7CE-1CD3-5059-B0AF-F2C47BA127E1

Figs 6, 21, 35

Metathrinca fopingensis Wang, Zheng & Li, 2000: 230. TL: China (Shaanxi: Foping County). TD: NKU.

#### Material examined.

***Holotype***: China: • ♂; Shaanxi Province, Foping County, Daguping Nature Reserve Station; 1000 m; 25 July 1985; leg. HH Li. ***Paratypes***: China: • 2♀; same data as holotype.

#### Additional material examined.

China: • 1♀; Chongqing, Mt. Jinfo, Daheba; 800–850 m; 16 Aug. 2011; leg. XC Du. • 1♀; Fujian, Wuyishan, Tongmu Village; 27.75°N, 117.63°E; 730 m; 11 Aug. 2020; leg. MJ Qi & XY Jin; slide no. TSY24432 • 1♀; Guizhou, Leigongshan, Fangxiang Township; 26.44°N, 108.27°E; 910 m; 2 Aug. 2018; leg. ML Zheng, JQ Deng & XJ Zhu. • 3♂14♀; Shaanxi, Taibai County, Huangbaiyuan Town; 33.48°N, 107.32°E; 1295 m; 19–22 July 2014; leg. JY Luo & Y Fei; slide nos. TSY24299♂, TSY24093♂ • 3♀; Shaanxi, Taibai County, Huangbaiyuan Town; 22 Aug. 2014; leg. KL Liu & Y Fei; slide no. TSY24427 • 3♂4♀; Shaanxi, Taibai County, Huangbaiyuan Town; 19–29 Aug. 2014; leg. KL Liu & JY Luo; slide no. TSY24032 • 2♀; Shaanxi, Taibai County, Huangbaiyuan; 33.48°N, 107.32°E; 1295 m; 1 July 2016; leg. H Wei & SS Wu; slide no. TSY24219 • 1♀; Shaanxi, Taibai County, Huangbaiyuan; 33.48°N, 107.32°E; 1295 m; 1 July 2016; leg. MY Cheng & YF Hou; slide no. TSY24001 • 1♂; Shaanxi, Taibai County, Huangbaiyuan; 33.81°N, 107.53°E; 1441 m; 16 Aug. 2020; leg. ML Li; slide no. TSY24334 • 1♀; Shaanxi, Taibai County, Huangbaiyuan; 33.81°N, 107.53°E; 1441 m; 15 Aug. 2020; leg. R Guo. • 3♀; Shaanxi, Hanzhong, Nanzheng County, Beiba Town; 32.62°N, 106.15°E; 849 m; 13 Aug. 2023; leg. S Ma, HL Yu & WQ Jing; slide nos. TSY24096, TSY24043, TSY24311 • 3♀; Shaanxi, Hanzhong, Nanzheng County, Liping Town; 32.86°N, 106.59°E; 1232 m; 12 July 2023; leg. S Ma, HL Yu & WQ Jing; slide no. TSY24416 • 1♀; Shaanxi, Houzhenzi; 33.88°N, 107.84°E; 1330 m; 24 July 2018; leg. WY Yang & JL Zhuang; slide no. TSY24004 • 1♂; Shaanxi, Huangbaiyuan; 33.37°N, 107.14°E; 1530 m; 18 July 2018; leg. YY Li & WX Yang. • 1♀; Shaanxi, Ningshan County, Xunyangba Town; 1400 m; 3 Aug. 2014; leg. HL Yu & KL Liu. • 1♀; Shaanxi, Ningshan County, Xunyangba Town; 1400 m; 4 Aug. 2014; leg. KL Liu & JY Luo. • 1♂; Shaanxi, Donghetai; 33.24°N, 107.30°E; 1400 m; 25 July 2018; leg. YY Li & T Liu. • 1♂1♀; Shaanxi, Donghetai; 33.24°N, 107.30°E; 1400 m; 26 July 2018; leg. YY Li & WX Yang • 1♂; Shaanxi, Hanzhong, Bashan Town; 32.73°N, 106.23°E; 1130 m; 23 July 2022; leg. WQ Jing & ZH Niu; slide no. TSY24014 • 1♀; Shaanxi, Huoditang Forest Farm; 33.15°N, 108.15°E; 1530 m; 28 July 2018; leg. YY Li & JL Zhuang. • 1♂; Shaanxi, Taibai County, Huangbaiyuan; 33.81°N, 107.51°E; 1300 m; 27 June 2014; leg. ZH Niu & YG Li; slide no. TSY24346 • 1♂; Sichuan, Maoxian County, Songpinggou; 32.09°N, 103.64°E; 2385 m; 4 July 2021; leg. S Yu, XJ Zhu & D Zhang; slide no. TSY24340 • 4♂; Sichuan, Wanglang, Baozi Valley; 32.91°N, 104.16°E; 2369 m; 20 July 2017; leg. MJ Qi & XF Yang; slide nos. TSY24215, TSY24208 • 1♂; Sichuan, Wanglang Hotel Hillside; 32.97°N, 104.10°E; 2569 m; 20 July 2017; leg. MJ Qi & XF Yang; slide no. ZAP24013 • 4♂; Sichuan, Wanglang, Changbai Valley; 32.93°N, 104.14°E; 2454 m; 14 July 2017; leg. MJ Qi & XF Yang; slide nos. TSY24218, TSY24370, TSY24209, TSY24146 • 1♂; Sichuan, Wanglang, Muyangchang; 32.97°N, 104.10°E; 2570 m; 19 July 2017; leg. MJ Qi & XF Yang; slide no. TSY24184 • 1♀; Sichuan, Baoxing County, Fengtongzhai; 30.57°N, 102.88°E; 1723 m; 27 July 2021; leg. S Yu, XJ Zhu & D Zhang; slide no. TSY24540.

#### Diagnosis.

Adult (Fig. 6). Wingspan 20.0–29.0 mm. *Metathrinca fopingensis* is diagnosed in the male genitalia by the apex of the uncus broadly concave at middle, forming two falcate lateral lobes spreading obliquely outward, the bilobed gnathos broadly mound-like, and the saccular process with base extending outward as a narrow rectangular band (Fig. 21); and in the female genitalia by the circular ostium bursae located in the middle on the posterior margin of the eighth sternum and with a sclerotized plate on each side, and the elliptical corpus bursae with a subrhombic signum (Fig. 35, 35a). It is similar to *M. tsugensis*, and the differences between them are stated in the diagnosis of *M. tsugensis*.

#### Distribution.

China (Chongqing, Fujian, Guizhou, Shaanxi, Sichuan).

### Metathrinca fortispina

Taxon classificationAnimaliaLepidopteraXyloryctidae

Li sp. nov.

303F51E1-64B9-5A0C-8148-2471F5A96A31

https://zoobank.org/F9AEED94-97DC-4076-8045-F145BFBD391C

Figs 7, 22, 36

#### Type material.

***Holotype***: China: • ♂; Guizhou Province, Heiwan, Mt. Fanjing; 530 m; 2 June 2022; leg. XP Wang; slide no. TSY25073. ***Paratypes***: China: • 1♂2♀; Guangxi, Mt. Daming; 1250 m; 27 May 2021; leg. LL Yang & YH Mou; slide nos. TSY25101♂, TSY25407♀.

#### Diagnosis.

The new species is similar to *M. ceromorpha* Meyrick, 1923 superficially. It can be separated from the latter in the male genitalia by the uncus narrowed from the base to a pointed apex, the subrectangular gnathos shorter than wide, and the phallus with one spine-shaped process before the apex; in *M. ceromorpha*, the uncus is subtrapezoidal, the gnathos is bilobed, and the phallus lacks a process before the apex (Clarke 1955: 447, pl. 222, fig. 2). The female genitalia are diagnosed by the eighth sternum with a rectangular plate on the posterior margin, and the posteriorly broadened antrum straight on the left margin and arched on the right margin.

#### Description.

Adult (Fig. 7). Wingspan 19.5–23.0 mm.

***Head***. Vertex and frons yellowish white. Labial palpus curved upward to level of vertex, apex acute; segments I and II brown on outer side, yellow on inner side, segments III whitish yellow. Male antenna with scape yellowish white, flagellum dark brown, finely annulated with yellow; female antenna with scape yellow, flagellum yellowish brown.

***Thorax***. Mesonotum and tegula whitish yellow. Forewing narrow at base, widened to obtusely produced apex, termen oblique; pale yellowish brown except brownish yellow between fold and dorsum, costal margin black in basal 1/4; subterminal and terminal lines yellow; fringe yellow. Hindwing and fringe yellowish white. Foreleg with femur brown, tibia and tarsus dark brown, tarsomeres yellowish brown at apices; midleg with femur white, tibia yellowish white, tarsus brown; hindleg white except tarsus brown.

***Abdomen***. Male genitalia (Fig. 22) with uncus subtriangular, broad at base, narrowed to strongly sclerotized, pointed apex. Gnathos subrectangular, shorter than wide, with dense protuberances. Tegumen subtrapezoidal, laterally extended to rounded anterior end. Valva narrowly elongate, uniform in width medially, narrowed to bluntly rounded apex distally; costal margin protruded at base; ventral margin concave at basal 2/5. Sacculus narrow at base, slightly widened to its outer margin, longer than 1/3 length of ventral margin of valva ventrally; saccular process irregularly L shaped, gradually widened to subapex, apex acute, with clustered dense fine spines distally. Juxta funnel-shaped. Saccus elongate triangular. Phallus ~ 3/5 length of valva, tapered to apex, with a spine-shaped process before apex; cornutus nearly as long as phallus, spiniform.

Female genitalia (Fig. 36, 36a). Papilla anales quadrate, setose. Apophyses posteriores ~ 1.3× length of apophyses anteriores. Eighth sternum with a strongly sclerotized rectangular plate at middle on posterior margin, setose posteriorly. Antrum broadened posteriorly, narrowed anteriorly, straight on left margin, smoothly arched on right margin posteriorly. Ductus bursae long, ~3× length of corpus bursae. Corpus bursae ovoid; signum oval, with a sclerotized transverse medial band (Fig. 36a).

#### Distribution.

China (Guangxi, Guizhou).

#### Etymology.

The specific epithet is derived from the Latin *fortis* and *spina*, referring to the strong preapical spine-shaped process of the phallus.

### Metathrinca graciliphalla

Taxon classificationAnimaliaLepidopteraXyloryctidae

Li sp. nov.

5C8F002F-1FF6-5049-8924-0B6CDD849525

https://zoobank.org/18458BB5-C19A-46FC-8963-6BE8EF5C109D

Figs 8, 23

#### Type material.

***Holotype***: China: • ♂; Hainan Province, Tianchi, Jianfengling; 18.73°N, 108.87°E; 787 m; 13–15 July 2015; leg. QY Wang, SR Li & MT Chen; slide no. TSY25420. ***Paratypes***: China: • 1♂; Hainan, Nanchahe, Bawangling; 600 m; 10 June 2007; leg. ZW Zhang & WC Li; slide no. TSY25250 • 1♂; Hainan, Diaoluoshan Nature Reserve; 18.43°N, 109.52°E; 922 m; 24 May 2015; leg. PX Cong, W Guan & S Hu; slide no. TSY25264 • 8♂; Hainan, Tianchi, Jianfengling; 18.73°N, 108.87°E; 787 m; 13–15 July 2015; leg. QY Wang, SR Li & MT Chen; slide no. TSY25226 • 1♂; Hainan, Limushan Forest Park; 19.10°N, 109.44°E; 607 m; 17 May 2015; leg. PX Cong, W Guan & S Hu; slide no. TSY25340 • 1♂; Hainan, Ledong County, Jianfengling Nature Reserve; 18.44°N, 108.52°E; 770 m; 28 May 2015; leg. PX Cong, W Guan & S Hu; slide no. TSY25295 • 3♂; Hainan, Ledong County, Jianfengling Nature Reserve; 18.74°N, 108.87°E; 770 m; 15 July 2014; leg. PX Cong, LJ Liu & S Hu; slide nos. TSY25299, TSY25300, TSY25301 • 2♂; Hainan, Ying’gezui, Ying’geling; 19.05°N, 109.56°E; 599 m; 29 July 2017; leg. X Bai, P Liu & S Yu; slide no. TSY25253 • 2♂; Hainan, Ledong County, Jianfengling; 18.75°N, 108.87°E; 810 m; 12, 15 May 2018; leg. P Liu, X Bai & S Yu; slide nos. TSY25244, TSY25248.

#### Diagnosis.

The new species can be distinguished from its congeners by the combination of the following male genital features: the subquadrate uncus is slightly produced outward lateroapically, the bilobed gnathos has each lobe strongly thumb shaped, the elongate triangular valva is straight on the costal margin and concave beyond basal 1/3 on the ventral margin, and the phallus is curved in C shape and longer than the valva.

#### Description.

Adult (Fig. 8). Wingspan 17.0–20.0 mm.

***Head***. Vertex and frons white. Labial palpus with segment I brown; segment II white, except brown in basal 1/3 dorsally and in basal 1/2 laterally; segment III white. Male antenna with scape brown, flagellum black.

***Thorax***. Mesonotum, tegula and forewing white. Forewing narrow at base, subparallel from near base to before obtusely rounded apex, termen slightly obliquely arcuate; subterminal line pale brown, dark yellowish brown at starting point on costal margin; terminal line pale yellowish brown, dark yellowish brown at starting point on costal margin; fringe white, with a black subbasal line, tipped with greyish brown. Hindwing and fringe white. Foreleg with femur white, tibia and tarsus brown, each tarsomere yellowish brown at apex; midleg white, tarsus brown, each tarsomere yellowish brown at apex; hindleg with femur white, distal four tarsomeres pale brown, white at apices.

***Abdomen***. Male genitalia (Fig. 23). Uncus subquadrate, apex shallowly concave in middle, slightly produced outward latero-apically, with a quadrate anterior emargination. Gnathos separated into two stout thumb-shaped lobes, with dense protuberances. Tegumen subtrapezoidal, laterally narrowed to anterior end. Valva elongate triangular, wide at base, narrowed to narrowly pointed apex; costal margin straight, with short setae; ventral margin concave beyond basal 1/3. Sacculus subtrapezoidal, almost reaching costal margin, < 1/4 length of ventral margin of valva posteriorly ventrally; saccular process weakly extended outward at base, extending obliquely outward and ventrad basally, slightly arched ventrad medially, with a cluster of spines along distal 1/2, denser towards apex, apically reaching costal margin before apex of valva. Juxta broad near base, distal 3/4 narrower, uniform in width. Saccus small, obtusely rounded anteriorly. Phallus slender, longer than valva, curved in broad C shape, tapering from base to before slightly enlarged apex.

Female unknown.

#### Distribution.

China (Hainan).

#### Etymology.

The specific epithet is derived from the Latin *gracil*- and the term *phallus*, referring to the slender phallus of the new species.

### Metathrinca intacta

Taxon classificationAnimaliaLepidopteraXyloryctidae

(Meyrick, 1938)

962C3D4B-9C72-5FE0-88F6-861FCF25970E

Figs 9, 24, 37

Ptochoryctis intacta Meyrick, In: Caradja and Meyrick 1938: 14. TL: China (Yunnan: Lijiang). TD: NHMUK.
Metathrinca intacta : Clarke, 1955: 446.

#### Material examined.

China: • 5♂3♀; Yunnan Province, Dali, Mt. Weibao; 25.18°N, 100.34°E; 2205 m; 30 July–3 Aug. 2014; leg. KJ Teng, W Guan, XC Wang & SR Liu; slide nos. TSY24585♂, TSY25415♂, TSY25416♀, TSY25418♂.

#### Diagnosis.

*Metathrinca intacta* can be distinguished by the gnathos with a small projection between the two lobes, and the narrow sacculus extended to the costal margin. It is similar to *M. argentea*, and the differences between them are stated in the diagnosis of *M. argentea*.

#### Redescription.

Adult (Fig. 9). Wingspan 21.0–28.0 mm.

***Head***. Vertex and frons white. Labial palpus curved upward, reaching vertex; segment I brown; segment II white, light brown on outer surface in basal 1/2; segment III white. Male antenna with scape brown, flagellum light brown; female antenna brown.

***Thorax***. Mesonotum and tegula greyish brown, tegula white in distal 1/2. Forewing narrow at base, widened and subparallel medially, slightly narrowed from beyond distal 1/4 to obtuse apex, termen obtusely oblique; white, with light brown scales along veins from base to apex; terminal line pale brown; fringe white, with a light grey subbasal line, distally slightly tipped with light grey. Hindwing and cilia white. Foreleg brown; midleg with femur brown, tibia white, tarsus greyish brown intermixed with yellow; hindleg with femur yellow, tibia white, tarsus yellowish brown.

***Abdomen***. Male genitalia (Fig. 24). Uncus trapezoidal, concave medially at apex, forming two small lateral lobes; lateral margin sclerotized and folded, concave inward medially. Gnathos divided into two strong, dome-shaped lobes, each lobe with dense protuberances; small obtuse projection between these lobes medially. Tegumen subtrapezoidal, shortly extended anterolaterally. Valva broad at base, gradually narrowed to obtusely rounded apex; costal margin slightly convex near base; ventral margin concave at basal 1/3, densely setose. Sacculus irregularly band-shaped, straight on outer margin, reaching costal margin, 1/5 length of ventral margin ventrally; saccular process not distinctly extended outward at base, curved in C shape, open to costal margin, with a line of spines from basal 1/5 to apex, distally with a cluster of spines reaching costal margin of valva. Juxta membranous, broad near base, narrowed from near base to apex. Saccus small, not conspicuously extended. Phallus slender, curved basally, inflated in distal 1/6.

Female genitalia (Fig. 37, 37a). Papilla anales subquadrate. Apophyses anteriores ~ 1/2 length of apophyses posteriores. Eighth sternum concave medially on posterior margin. Ostium bursae circular, located at middle on posterior margin of eighth sternum. Antrum elongate, uniformly narrow and straight posteriorly, roundly dilated anteriorly. Ductus bursae membranous, slightly widening toward corpus bursae. Corpus bursae elliptical; signum rhomboidal, more or less cross-shaped, with a sclerotized transverse band in middle, situated near entrance of corpus bursae (Fig. 37a).

#### Distribution.

China (Yunnan).

### Metathrinca licina

Taxon classificationAnimaliaLepidopteraXyloryctidae

Li sp. nov.

7DCD8C58-811B-5D57-9B5F-699C1E411F0F

https://zoobank.org/A9E30EC1-8684-4785-B469-DE925433D543

Figs 10, 25

#### Type material.

***Holotype***: China: • ♂; Hubei Province, Wufeng County, Houhe Nature Reserve; 30.12°N, 110.67°E; 1100 m; 11 July 1999; leg. HH Li et al.; slide no. TSY24504.

#### Diagnosis.

The new species can be distinguished from its congeners by the bilobed gnathos with a large triangular membranous projection between the two lobes, the auriform valva apically produced into a papillary process, and the saccular process extending far beyond the costal margin of the valva.

#### Description.

Adult (Fig. 10). Wingspan 20.0 mm.

***Head***. Vertex and frons yellowish white (slightly erased). Labial palpus curved upward, not reaching level of vertex; segments I and II yellowish brown, segment III white.

***Thorax***. Mesonotum and tegula white (erased). Forewing narrow at base, gradually widened to triangularly produced apex, apex blunt, termen oblique; white; terminal and subterminal lines yellowish brown; fringe white, with a brown subbasal line. Hindwing and fringe white, tinged with yellow. Foreleg brown; midleg yellowish brown, tarsus dark brown; hindleg with femur yellowish brown, tibia and tarsus white, tarsomeres brown at apices.

***Abdomen***. Male genitalia (Fig. 25). Uncus wide basally, narrowed medially, widened distally; apex concave medially, laterally produced into triangular lobes. Gnathos bilobed, each lobe thumb-shaped, rounded apically, with tubercles; same sized triangular medial projection between the two lobes. Tegumen subtrapezoidal, extended anterolaterally. Valva broad auriform, apex produced to a relatively large papillary process; costal margin concave at base, convex before apex; ventral margin smoothly arched. Sacculus extending to costal margin of valva, widened and as long as 1/3 length of ventral margin of valva ventrally; saccular process narrowly and triangularly extended outward at base, curved in U shape basally, tapering to a pointed apex far beyond costal margin of valva, with a line of spines from basal 1/6 becoming denser to apex. Juxta narrowed from near base, bifurcated from basal 1/3 into two gradually narrowed lobes. Saccus small, triangular. Phallus ~ 3/4 length of valva, slightly curved, tapered from base to apex.

Female unknown.

#### Distribution.

China (Hubei).

#### Etymology.

The specific epithet is derived from the Latin *licinus*, referring to the strongly up-curved saccular process of the male genitalia.

### Metathrinca lingugnathos

Taxon classificationAnimaliaLepidopteraXyloryctidae

Li sp. nov.

5C78D4BB-7EB5-550B-9B05-410DF3572CBB

https://zoobank.org/0170E485-AC8B-4F82-BAC8-830171E61D85

Figs 11, 26, 38

#### Type material.

***Holotype***: China: • ♂; Hainan Province, Ledong County, Jianfengling; 18.75°N, 108.87°E; 810 m; 13 June 2018; leg. P Liu, X Bai & S Yu; slide no. TSY25419. ***Paratypes***: China: • 5♂2♀; Hainan, same data as holotype, except dated 12–16 June 2018; slide nos. TSY24181♂, TSY24182♀, TSY24244♂, TSY24308♂, TSY24325♂, TSY25398♀, TSY25400♀ • 13♂; Hainan, Ledong County, Jianfengling Nature Reserve; 18.87°N, 108.87°E; 770 m; 13–17 July 2014; leg. PX Cong, W Guan & S Hu; slide nos. TSY24521, TSY25303, TSY25308 • 22♂3♀; Hainan, same locality; 18.44°N, 108.52°E; 770 m; 28 May–5 June 2015; leg. PX Cong, W Guan & S Hu; slide nos. TSY24193♂, TSY24194♂, TSY24196♂, TSY24197♂, TSY24198♂, TSY24229♂, TSY24263♂, TSY24344♂, TSY24345♂, TSY24352♂, TSY24384♂, TSY24400♂, TSY24403♂, TSY24423♀, TSY24444♀, TSY25223♂, TSY25283♂, TSY25291♂, TSY25294♂, TSY25296♂ • 15♂3♀; Hainan, Tianchi, Jianfengling; 18.73°N, 108.87°E; 787 m; 12–16 July 2015; leg. QY Wang, SR Li & MT Chen; slide nos. TSY24519♂, TSY25227♂ • 2♂; Hainan, same data; 7–8 July 2015; leg. X Bai, SN Qian & WD Qi; slide no. TSY24282 • 1♂; Guangdong, Shaoguan, Nanling Mountains; 7–14 July 2007; leg. M Wang et al.; slide no. TSY24289 • 4♂; Guangdong, Ruyuan Yao Autonomous County, Nanling Mountains; 24.91°N, 113.03°E; 830 m; 10–11 July 2023; leg. LL Yang; slide nos. TSY25233, TSY25234 • 1♂; Guangxi, Jinxiu County, Dayaoshan National Nature Reserve, Hekou Protection Station; 24.14°N, 110.09°E; 823 m; 20 July 2015; leg. MJ Qi & SN Zhao; slide no. TSY25239 • 1♂; Guizhou, Jiangkou County, Taiping Town; 27.85°N, 108.78°E; 511 m; 14 July 2019; leg. MR Xing, BX Zhao & H Sun; slide no. TSY24343 • 1♂; Guizhou, Shiqian County, Mt. Foding; 27.35°N, 108.16°E; 618 m; 17 July 2019; leg. MR Xing, BX Zhao & H Sun; slide no. TSY25238 • 10♂2♀; Hubei, Wufeng County, Houhe; 1000 m; 12 July 1999; leg. HH Li et al.; slide nos. TSY24510♂, TSY24553♂, TSY24581♂, TSY24592♀, TSY24619♂, TSY25185♀, TSY25186♂, TSY25187♂, TSY25188♂ • 5♂; Zhejiang, Jiangshan City, Laofoyan Village; 28.37°N, 118.68°E; 413 m; 3 July 2017; leg. ZG Zhang, YY Jia & J Li; slide nos. TSY24285, TSY24412, TSY24465.

**Figures 38–42.**
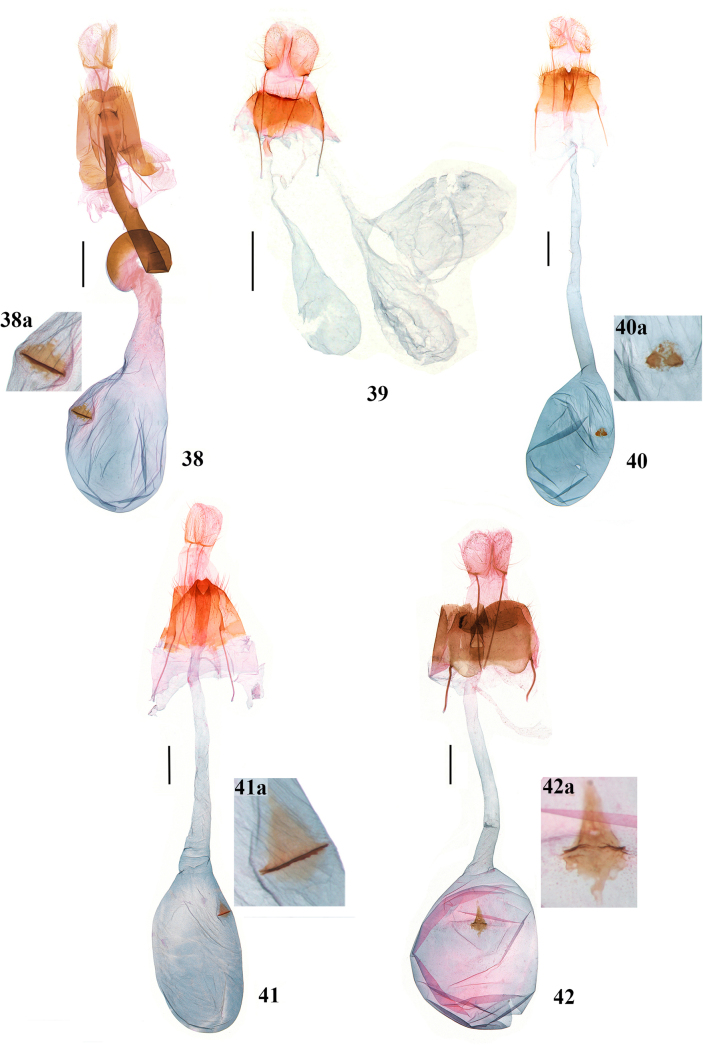
Female genitalia of *Metathrinca* spp. **38**. *M. lingugnathos* Li, sp. nov., slide no. TSY25398, paratype; **39**. *M. meihuashana* Wang, Zheng & Li, 2000, slide no. TSY25115; **40**. *M. nigrivenosa* Li, sp. nov., slide no. TSY25151, paratype; **41**. *M. punctuatella* Li, sp. nov., slide no. TSY25375, paratype; **42**. *M. tsugensis* (Kearfott, 1910), slide no. TSY25417. **38a–42a**. Enlarged view of signum. Scale bars: 0.5 mm.

#### Diagnosis.

The new species is similar to *M. mutativalvis* Li, 2025 in the male genitalia. It can be distinguished from the latter by the uncus with two apex-pointed distal lobes, and the saccular process extending outward and almost reaching the apex of the valva; in *M. mutativalvis*, the uncus has two apex-rounded distal lobes, and the saccular process is curved obliquely dorsad and terminates at ~ 3/4 of the valva (Zuo et al. 2025: 156, fig. 2B).

#### Description.

Adult (Fig. 11). Wingspan 20.5–28.0 mm.

***Head***. Vertex and frons white. Labial palpus curved upward, reaching below vertex; yellowish brown, paler in female. Male antenna with scape dark brown on dorsal surface, earth yellow on ventral surface, flagellum black; female antenna with scape brown dorsally, whitish yellow ventrally, flagellum dark brown.

***Thorax***. Mesonotum, tegula and forewing white. Forewing narrow at base, subparallel from near base to before broadly obtuse apex, termen slightly oblique; subterminal and terminal lines pale brown, dark yellowish brown at starting point on costal margin, parallel, between subterminal and terminal lines placed transversely subrectangular greyish spots of raised scales; fringe white, with a distinct yellowish brown subbasal line, tipped with pale brown. Hindwing and fringe white. Foreleg with femur white, tibia and tarsus dark brown, tarsomeres yellowish brown at apices; midleg with femur white, tibia brown, tarsus dark brown, tarsomeres yellowish brown at apices; hindleg white, distal four tarsomeres brown, white apically.

***Abdomen***. Male genitalia (Fig. 26). Uncus subtrapezoidal; apex concave medially, forming a pair of small sclerotized lateral lobes pointed at apex; with paired longitudinal projections medially on ventral surface. Gnathos sublingulate distally, strongly sclerotized, with dense protuberances. Tegumen subtrapezoidal, laterally extended to rounded anterior end. Valva wide at base, slightly narrowed to rounded apex; costal margin slightly concave, with long setae in distal 3/4; ventral margin shallowly concave at basal 1/3, with sparse setae. Sacculus narrowed to 1/3 from costal margin, < 1/3 length of ventral margin of valva ventrally; saccular process extended outward as a narrow bar at base, curved downward to ventral margin, forming a U shape basally, then extending outward to before apex of valva, thickening from near base to distal 1/3, with a brush of spines from near base to apex, with a strongly sclerotized apical spine. Juxta narrowed from near base to apex, distal 2/3 bilobed. Saccus subtriangular. Phallus strongly sclerotized, slightly shorter than 4/5 length of valva, tubular from basal 1/4 to rounded apex; cornutus spiniform.

Female genitalia (Fig. 38, 38a). Papilla anales subovate, setose. Apophyses posteriores ~ 2.5× length of apophyses anteriores. Eighth sternum with a sclerotized subrectangular plate posteriorly, shallowly concave medially on posterior margin. Ostium bursae circular, located at posterior 1/3 of eighth sternum, with paired subtriangular sclerites posterolaterally; ductus bursae elongate, sclerotized in posterior 2/3, coiled into a loop before middle, slightly widened and membranous toward corpus bursae anteriorly. Corpus bursae ovoid; signum subovate, transversely with a sclerotized erect ridge (Fig. 38a).

#### Distribution.

China (Guangdong, Guangxi, Guizhou, Hainan, Hubei, Zhejiang).

#### Etymology.

The specific epithet is derived from the Latin *lingu*- and the term gnathos, referring to the distal tongue-like gnathos in the male genitalia.

### Metathrinca meihuashana

Taxon classificationAnimaliaLepidopteraXyloryctidae

Wang, Zheng & Li, 2000

D1A0640E-C608-5706-84AC-45031936869E

Figs 12, 27, 39

Metathrinca meihuashana Wang, Zheng & Li, 2000: 232. TL: China (Fujian: Mt. Meihua). TD: NKU.

#### Material examined.

***Holotype***: China: • ♂; Fujian Province, Mt. Meihua; 19 July 1988.

#### Additional material examined.

China: • 1♀; Hainan, Wuzhishan City, Shuiman Township; 18.88°N, 109.65°E; 640 m; 28 Oct. 2016; leg. X Bai, SN Qian & WD Qi; slide no. TSY25115 • 6♂2♀; Hainan, Ying’geling; 620 m; 12 April 2010, 21 April 2010, 7 May 2010, 9 May 2010, 23 May 2010; leg. BB Hu & J Zhang; slide nos. TSY25075♂, TSY24245♂ • 8♀; Hainan, Ying’geling, Ying’gezui; 19.05°N, 109.56°E; 599 m; 27 July 2017, 29 July 2017, 1 Aug. 2017; leg. X Bai, P Liu & S Yu; slide nos. TSY24232, TSY24424, TSY24462, TSY24463 • 3♀; Hainan, Wuzhishan City, Shuiman Township; 18.88°N, 109.67°E; 766 m; 5 July 2015, 7 July 2015; leg. QY Wang, SR Li & MT Chen. • 2♀; Hainan, same locality; 27 Feb. 2016; leg. QY Wang, SR Li & SN Zhao. • 1♀; Hainan, same locality; 3 Aug. 2016; leg. X Bai, SN Qian & WD Qi; slide no. TSY24442 • 5♀; Hainan, Wuzhishan Nature Reserve; 18.53°N, 109.39°E; 742 m; 18–21 May 2015; leg. PX Cong, W Guan & S Hu; slide no. TSY24234 • 4♂; Hainan, Wuzhishan Nature Reserve; 740 m; 12–14 April 2009; leg. Q Jin & BB Hu; slide no. TSY25076 • 3♂; Hainan, Wuzhishan National Park; 18.88°N, 109.67°E; 766 m; 9 Jan. 2016, 12 Jan. 2016; leg. KJ Teng, X Bai & MT Chen. • 3♂; Fujian, Huboliao Nature Reserve Ecological Station; 24.53°N, 117.24°E; 364 m; 24 July 2020; leg. MJ Qi & XY Jin; slide nos. TSY24159, TSY24258, TSY24388 • 1♂; Fujian, Nanjing County, Zijingshan; 24.49°N, 117.36°E; 175 m; 26 July 2020; leg. MJ Qi & XY Jin; slide no. TSY24172 • 1♂; Fujian, Longyan, Mt. Meihua, Yunshan Management Station; 25.32°N, 116.94°E; 443 m; 31 July 2020; leg. MJ Qi & XY Jin. • 2♂; Guangxi, Shangsi, Mt. Pinglong; 510 m; 6 April 2002; leg. SL Hao & HJ Xue; slide nos. TSY24546, TSY25212 • 1♂1♀; Guizhou, Xishui, Sanchahe Town; 28.48°N, 106.42°E; 767 m; 29–30 June 2019; leg. MR Xing, BS Zhao & H Sun; slide no. TSY24425♀.

#### Diagnosis.

*Metathrinca meihuashana* is distinguished in the male genitalia by the uncus strongly separated into two lobes from near the base, the broad subcircular gnathos covered with dense granules, the stout saccular process curved in U-shape and open towards the base, and the juxta bifurcated into slender branches distally.

#### Description.

Adult (Fig. 12). Wingspan 12.0–18.5 mm. ***Head*** and ***thorax*** as previously described for male by Wang et al. (2000).

***Abdomen***. Male genitalia (Fig. 27). As previously described by Wang et al. (2000).

Female genitalia (Fig. 39). Papilla anales broad and short, setose. Apophyses posteriores ~ 1.3× length of apophyses anteriores. Eighth sternum strongly sclerotized on posterior margin, forming a narrow band. Antrum short, narrow. Ductus bursae slender, membranous, shorter than corpus bursae; ductus seminalis arising from posterior 2/5 of ductus bursae; accessory bursae membranous, slightly larger than corpus bursae. Corpus bursae membranous, without signum.

#### Distribution.

China (Hainan, Fujian, Guangxi, Guizhou).

### Metathrinca mutativalvis

Taxon classificationAnimaliaLepidopteraXyloryctidae

Li, 2025

74C20470-1C84-50CA-88C4-76C6F8845A18

Metathrinca mutativalvis Zuo, Tang & Li, 2025: 155. TL: China (Sichuan: Mt. E’mei). TD: NKU.

#### Type materials and descriptions.

See Zuo et al. (2025).

#### Distribution.

China (Hainan, Hubei, Sichuan).

### Metathrinca nigrivenosa

Taxon classificationAnimaliaLepidopteraXyloryctidae

Li sp. nov.

321D4CD3-81D3-52FB-8D37-41C1663A70E4

https://zoobank.org/303C4637-5995-4AD1-BA15-352099F00629

Figs 13, 28, 40

#### Type material.

***Holotype***: China: • ♂; Yunnan Province, Taiyanghe Nature Reserve; 1450 m; 18 May 2014; leg. ZG Zhang; slide no. TSY24488. ***Paratype***: China: • 1♀; Xizang, Medog County, Beibeng Township, Delgong Village; 26 May–4 June 2021; leg. HL Han; slide no. TSY25151.

#### Diagnosis.

The new species is similar to *M. rosaria* (Meyrick, 1907) in the male genitalia. It can be distinguished by the forewing with black scales along the veins from basal 1/3 to the yellowish brown subterminal line, whereas the forewing of the latter species lacks black scales along the veins and the subterminal line consists of several black dots. The male genitalia of the new species are not distinguishable from those of *M. rosaria*. The female genitalia are distinguished from those of the other species by the slender ductus bursae > 2× the length of the corpus bursae, and the subtriangular signum without a medial ridge.

The new species is also similar to *M. punctuatella* Li, sp. nov. in the male genitalia, and the differences between the two species are stated in the diagnosis of the latter species.

#### Description.

Adult (Fig. 13). Wingspan 28.0–29.0 mm.

***Head***. Vertex and frons snowy white. Labial palpus curved upward to level of vertex; dark brown, segment II with basal 1/4 white on inner surface. Antenna with scape white ventrally, dark brown dorsally; flagellum black, narrowly ringed with dark yellowish brown.

***Thorax***. Mesonotum and tegula cream white. Forewing narrow at base, widened to before triangularly produced apex, termen oblique; white, with scattered black scales from basal 1/3 to subterminal line along veins; subterminal line light yellowish brown; fringe white, with a pale brown subbasal line, tipped with light brown. Hindwing white; fringe white, with a pale greyish brown subbasal line. Foreleg with femur white, tibia and tarsus dark brown, tarsomeres white at apices; mid- and hindlegs white, tarsi brown, tarsomeres white at apices.

***Abdomen***. Male genitalia (Fig. 28). Uncus elongate, wide at base, narrowed to apex; apex concave at middle, forming two small triangular lobes extending obliquely outward. Gnathos rectangular, sclerotized, slightly wider basally, blunt at apex, with dense protuberances. Tegumen widest medially, greatly extended laterally, uniformly narrow to before rounded apex. Valva subparallel from base to 3/4, triangularly narrowed from distal 1/4 to pointed apex, setose; costal margin slightly convex at distal 1/4; ventral margin slightly concave at basal 1/3, then obtusely arched to apex. Sacculus subtrapezoidal, extending to ~ 1/3 from costal margin, 1/5 length of ventral margin of valva ventrally; saccular process narrowly extended outward at base, curved basally, slightly exceeding apex of valva, with a line of dense spines from near base to apex, denser apically. Juxta narrowed from near base to apex, bilobed distally. Saccus triangular. Phallus slightly curved, weakly sclerotized, ~ 1/2 length of valva, tapered to apex.

Female genitalia (Fig. 40, 40a). Papilla anales subquadrate, with short setae. Apophyses anteriores ~ 1.6× length of apophyses posteriores. Eighth sternum with posterior margin sclerotized and setose, deeply concave in obtuse V shape medially. Ostium bursae circular, situated at posterior 1/3 of eighth sternum. Antrum short, boundary with ductus bursae indistinct. Ductus bursae long, > 2× length of corpus bursae; ductus seminalis arising from posterior 1/5 of ductus bursae. Corpus bursae elliptical; signum subtriangular (Fig. 40a).

#### Distribution.

China (Xizang, Yunnan).

#### Etymology.

The specific epithet is derived from the Latin *nigri*- and *venosus*, referring to the forewing with black scales along veins from basal 1/3 to the subterminal line.

### Metathrinca punctuatella

Taxon classificationAnimaliaLepidopteraXyloryctidae

Li sp. nov.

63CF02A5-DE59-5393-AE9B-C34FCE4D38C5

https://zoobank.org/7891426F-F9DE-44B6-93D3-EDF1006C6F63

Figs 14, 29, 41

#### Type material.

***Holotype***: China: • ♂; Hainan Province, Qiongzhong County, Limushan Nature Reserve; 19.17°N, 109.74°E; 640–700 m; 4 May 2014; leg. TT Liu, W Guan & XM Hu; slide no. TSY25421. ***Paratypes***: China: • 5♂4♀; Hainan, Ledong County, Jianfengling Nature Reserve; 18.44°N, 108.52°E; 770 m; 28 May–5 June 2015; leg. PX Cong, W Guan & S Hu; slide nos. TSY24203♂, TSY24267♂, TSY24291♂, TSY25396♂, TSY25397♀ • 1♀; Hainan, Ledong County, Jianfengling; 18.75°N, 108.87°E; 810 m; 13 June 2018; leg. P Liu, X Bai & S Yu. • 3♀; Hubei, Yingshan County, Taohuachong; 30.99°N, 116.02°E; 635 m; 23 June 2014; leg. W Guan & MQ Yang; slide no. TSY25375 • 2♂; Hubei, Yingshan County, Mt. Wujia; 31.53°N, 115.47°E; 800 m; 28 June 2014; leg. W Guan & MQ Yang. • 1♂; Taiwan, Taipei, Taoyuan City, Mt. Lala; 1800 m; 1 Aug. 2006; leg. HH Li & XC Du • 1♂; Zhejiang, Mt. Tianmu, Xianren Peak; 1500 m; 18 Aug. 1999; leg. HH Li et al.; slide no. TSY24601.

#### Diagnosis.

The new species is similar to *M. rosaria* (Meyrick, 1907) and *M. nigrivenosa* sp. nov. in the male genitalia. It can be distinguished from *M. rosaria* by the forewing having a yellowish brown subterminal line, which consists of several black dots in *M. rosaria*. The gnathos is deeply concave in V shape distally and the saccular process is curved to ventral margin of the valva basally in U shape in the new species; whereas the apex of the gnathos is only slightly notched at middle and the saccular process is slightly curved to above the ventral margin basally in *M. rosaria* (Clarke, 1955: 449, pl. 223, figs 4, 4a, 4b). The new species can be distinguished from *M. nigrivenosa* sp. nov. in the male genitalia by the uncus with two rounded apical lobes, and the valva with the ventral margin nearly straight in basal 3/5 and obliquely straight in distal 2/5; in *M. nigrivenosa* sp. nov., the uncus has two small triangular apical lobes, and the ventral margin of the valva is slightly concave at basal 1/3 and then obtusely arched to the apex.

#### Description.

Adult (Fig. 14). Wingspan 20.5–27.5 mm.

***Head***. Vertex and frons white. Labial palpus with segment I white on inner surface, brown on outer surface; segment II white, brown in basal 1/2 dorsally; segment III white, apex acute. Male antenna with scape brown, flagellum dark brown; female antenna with scape white dorsally, brown ventrally, flagellum dark brown dorsally, yellowish brown ventrally.

***Thorax***. Mesonotum and tegula white. Forewing narrow at base, subparallel from near base to before obtusely rounded apex, termen oblique; white, with sparse brown scales distally; pale brown spot placed at end of cell, diffused outward to posterior end of subterminal line; subterminal and terminal lines yellowish brown, between them placed several pale ovate yellowish-brown spots of raised, transversely widened scales; fringe white, with a dark brown subbasal line, tipped with pale brown distally. Hindwing and fringe cream white. Foreleg with femur yellowish white, tibia and tarsus dark brown, yellowish brown at apex of each tarsomere; midleg white, tarsus brown, yellowish brown at apex of each tarsomere; hindleg yellowish white, distal four tarsomeres pale brown, white at apices.

***Abdomen***. Male genitalia (Fig. 29). Uncus nearly trapezoidal, narrowly and weakly sclerotized along longitudinal midline; apex slightly concave medially, forming a pair of short rounded lobes. Gnathos heavily sclerotized, subequal in width, concave in broad V shape apically, with dense protuberances. Tegumen trapezoidal posteriorly, extending obliquely outward anterolaterally. Valva narrowly elongate, basal 2/3 parallel sided, slightly narrowed from distal 1/3 to before triangularly produced apex; costal margin slightly convex at distal 1/5; ventral margin nearly straight in basal 3/5, straight but oblique in distal 2/5. Sacculus subtrapezoidal, extending to 1/5 from costal margin, ~ 1/5 length of ventral margin of valva ventrally; saccular process slightly extended outward at base, arched downward to ventral margin, then narrowed to beyond apex of valva, U-shaped basally, with a cluster of spines becoming denser from near base to apex. Juxta wide near base, narrowed distally, bifurcated from basal 1/2 into two gradually narrowed lobes. Saccus small, subtriangular. Phallus ~ 1/2 length of valva, tapering to apex from basal 1/3.

Female genitalia (Fig. 41, 41a). Papilla anales ovate, with short setae. Apophyses anteriores slightly longer than 2/3 length of apophyses posteriores. Eighth tergum straight on posterior margin; eighth sternum medially extended posteriorly, with a small, shallow notch at middle. Ostium bursae small and circular, situated near posterior margin of eighth sternum. Antrum tubular, without clear boundary from ductus bursae. Ductus bursae slightly widened toward corpus bursae; ductus seminalis arising from posterior 1/4 of ductus bursae. Corpus bursae large, elongate elliptical, ~ 2/3 length of ductus bursae; signum small, subrhomboidal in posterior 1/3, with a sclerotized transverse ridge at anterior 1/4, which bears teeth (Fig. 41a).

#### Distribution.

China (Hainan, Hubei, Taiwan, Zhejiang).

#### Etymology.

The specific epithet is derived from the Latin *punctuatus* and -*ella*, referring to the small brown spot at the end of the cell on the forewing.

### Metathrinca sublunata

Taxon classificationAnimaliaLepidopteraXyloryctidae

Li sp. nov.

35CB04C9-8CBE-56D9-922E-9B492B8CD6A4

https://zoobank.org/C53A20CC-B0F1-430B-8C2B-135114305ADD

Figs 15, 30

#### Type material.

***Holotype***: China: • ♂; Hainan Province, Wuzhishan Nature Reserve; 18.53°N, 109.39°E; 742 m; 20 May 2015; leg. PX Cong, W Guan & S Hu; slide no. TSY25277. ***Paratypes***: China: • 1♂; Hainan, same data as holotype, except dated 21 May 2015; slide no. TSY25269 • 2♂; Hainan, Diaoluoshan National Nature Reserve; 18.43°N, 109.52°E; 922 m; 25 May 2015; leg. PX Cong, W Guan & S Hu; slide nos. TSY24268♂, TSY24323♂ • 1♂; Hainan, Wuzhishan City, Shuiman Township, Lizu Grand Hall; 18.88°N, 109.67°E; 776 m; 2 July 2016; leg. X Bai, SN Qian & WD Qi; slide no. TSY25334 • 3♂; Hainan, Diaoluoshan National Nature Reserve; 18.43°N, 109.52°E; 922 m; 26 May 2015; leg. PX Cong, W Guan & S Hu; slide no. TSY25272.

#### Diagnosis.

The new species is slightly similar to *M. memnon* Meyrick, 1914. Externally, *M. memnon* has a dark perpendicular medial streak on the forewing which is not present in *M. sublunata*. In the male genitalia, the new species is distinguished by the uncus beak-like with a pointed apex in lateral view, and the valva is smooth near the base; in *M. memnon*, the apex of the uncus is concave medially, and the valva has a raised elongate process extending downward from beyond the basal concavity of the costal margin (Meyrick 1914: 778; Clarke 1955: 449, pl. 223, figs 2, 2a, 2b).

#### Description.

Adult (Fig. 15). Wingspan 15.0–17.5 mm.

***Head***. Vertex and frons white. Labial palpus curved upward exceeding vertex; earth yellow. Male antenna with scape brownish yellow, flagellum dark brown.

***Thorax***. Mesonotum, tegula and forewing white. Forewing narrow at base, subparallel from near base to before distal 1/4, then narrowed to apex, termen obtusely oblique; subterminal line yellow; fringe white, with a pale brown subbasal line. Hindwing and fringe white, tinged with yellow. Foreleg brown, tibia and tarsus dark brown; midleg with femur yellowish white, tibia white, tarsus pale brown; hindleg femur yellowish brown, tibia and tarsus white, except brown at apices of distal four tarsomeres.

***Abdomen***. Male genitalia (Fig. 30). Uncus subtriangular, wide at base, gradually narrowed to pointed apex, distally beak-like in lateral view. Gnathos sublingulate, narrowed to rounded apex, with dense protuberances. Tegumen subtrapezoidal posteriorly, anterolaterally extended and narrowed to narrowly rounded anterior end. Valva wide at base, slightly narrowed to rounded apex, setose; costal margin concave near base; ventral margin concave at basal 2/5. Sacculus elongate subelliptical, extending to 1/3 from costal margin, as long as 2/5 length of ventral margin of valva ventrally; saccular process shortly extended outward as a columnar process at base, sublunate in shape, narrowed basally, widened medially, gradually narrowed to acute apex, bent inward, bearing dense spines from near base to apex, becoming denser and clustered distally. Juxta widest at middle, slightly narrowed to apex, sharply narrowed from middle to base, forming a clubbed basal process. Saccus small, triangular. Phallus straight, tapered from broad base to apex, ~ 2/3 length of valva.

Female unknown.

#### Distribution.

China (Hainan).

#### Etymology.

The specific epithet is derived from the Latin *sublunatus*, referring to the shape of the saccular process.

### Metathrinca tsugensis

Taxon classificationAnimaliaLepidopteraXyloryctidae

(Kearfott, 1910)

D255F681-733D-5506-8F4A-E6EF6735539E

Figs 16, 31, 42

Ptochoryctis tsugensis Kearfott, 1910: 347. TL: Japan (two syntypes reared in New Jersey, USA on host plants imported from Japan). TD: NHMUK (depository status unconfirmed).
Metathrinca tsugensis : Moriuti in Inoue et al. 1982, 1: 259, 2: 208.

#### Material examined.

China: • 1♂; Anhui Province, Mt. Jiuhua, Jiuhuajie; 9 Aug. 2004; leg. JS Xu & JL Zhang; slide no. TSY25184 • 2♂; Anhui, Huoshan County, Mozitan; 31.17°N, 116.17°E; 12 Aug. 2004; leg. JS Xu & JL Zhang; slide nos. TSY25117, TSY25126 • 2♂; Chongqing, Youyang County, Mt. Jinyin; 660 m; 25 July 2012; leg. YH Sun & LJ Xu. • 1♀; Chongqing, Qianjiang, Wuli Township; 870 m; 24 July 2012; leg. J Zhang & LJ Xu. • 2♂; Fujian, Mt. Wuyi, Tongmu Village; 29 Aug. 2012, 2 Sep. 2012; leg. ZB Wang; slide nos. TSY24511, TSY24604 • 1♂; Fujian, Mt. Wuyi, Tongmuguan; 27.81°N, 117.71°E; 1090 m; 13 Aug. 2020; leg. MJ Qi & XY Jin. • 2♀; Guizhou, Chishui, Suoluo; 390 m; 27 May 2000; leg. LY Du. • 1♂; Guizhou, Libo County, Limingguan Shui Township, Pobao; 740 m; 20 July 2015; leg. MQ Yang & G-E Lee; slide no. TSY25171 • 1♂; Guangdong, Lianzhou, Mt. Dadong; 650 m; 21 June 2004; leg. DD Zhang; slide no. TSY24603 • 1♂; Henan, Tongbai, Shuiliandong; 300 m; 26 May 2000; leg. HL Yu. • 2♂; Hubei, Yingshan County, Taohuachong; 30.99°N, 116.03°E; 635 m; 23, 25 June 2014; leg. W Guan & MQ Yang. • 1♂; Hunan, Xinning County, Shunhuangshan Nature Reserve, Xiangshanjiang; 26.42°N, 111.01°E; 921 m; 4 Aug. 2020; leg. H Sun, XH Zuo & ZL Tao; slide no. TSY25127 • 1♀; Shaanxi, Houzhenzi; 33.88°N, 107.84°E; 1330 m; leg. WX Yang & JL Zhuang. • 1♂; Zhejiang, Mt. Jiulong; 6 Aug. 2011; leg. XB Fu; slide no. TSY25312 • 1♀; Zhejiang, Suichang, Mt. Jiulong, Huangshayao Township; 28.42°N, 118.82°E; 360 m; 24–27 May 2017; leg. SN Qian & G-E Lee. • 5♂3♀; Zhejiang, Jiangshan City, Shuangxikou Township, Laofoyan Village; 28.35°N, 118.68°E; 424 m; 7–9 Aug. 2016; leg. QY Wang, MQ Yang & P Liu; slide nos. TSY25205♂, TSY25417♀ • 24♂3♀; Zhejiang, Quzhou City, Datou Village; 29.10°N, 118.45°E; 707 m; 5–12 Aug. 2018; leg. S Yu, MR Xing & C Liu; slide no. TSY24518♂ • 1♂; Zhejiang, Kaihua County, Mt. Gutian; 29.14°N, 118.07°E; 416 m; 13 Aug. 2018; leg. S Yu, MR Xing & C Liu. • 2♂; Zhejiang, Jingning County, Wangdongyang Wetland Reserve; 27.41°N, 119.38°E; 1172 m; 16 Aug. 2018; leg. S Yu, MR Xing & C Liu; slide no. TSY25096.

#### Diagnosis.

Adult (Fig. 16). Wingspan 15.0–23.0 mm. *Metathrinca tsugensis* is distinguished in the male genitalia by the uncus concave at apex, the gnathos strongly bilobed, and the saccular process triangularly extended outward at base, and curved in C shape open to the costal margin (Fig. 31); in the female genitalia by the ostium bursae situated at the anterior 1/3 of the eighth sternum, and the ovate corpus bursae with a subrhomboidal signum placed at posterior 1/3 (Fig. 42, 42a).

#### Distribution.

China (Anhui, Chongqing, Fujian, Guangdong, Guizhou, Henan, Hubei, Hunan, Jiangxi, Shaanxi, Yunnan, Zhejiang), Japan.

### Metathrinca zhengi

Taxon classificationAnimaliaLepidopteraXyloryctidae

Li, 2025

D7254CE1-D320-5183-A9DB-1335F619A25A

Metathrinca zhengi Zuo, Tang & Li, 2025: 157. TL: China (Sichuan: Mt. E’mei). TD: NKU.

#### Type materials and descriptions.

See Zuo et al. (2025).

#### Distribution.

China (Chongqing, Guangdong, Hainan, Sichuan, Yunnan).

## Discussion

Most species in *Metathrinca* share a broad forewing medially subparallel sided and apically being either obtusely rounded or triangularly produced. They are extremely similar superficially, making it difficult to distinguish and to pair up individuals from the collected specimens. However, at the specific level, those species currently placed in *Metathrinca* generally exhibit strong diagnostic characters. Species identification mostly relies on the male genital features, including the uncus bilobed or not bilobed distally, the gnathos strongly separated into two lobes or only bilobed distally, and the shape of both the sacculus and the almost invariably spinous saccular process.

Our study focuses on the taxonomy of *Metathrinca* in China. However, there are several species not included in this study due to each having only a single specimen, which is insufficient to support a formal species description. But at least it indicates the existence of more undetermined species in China, and the species diversity within China suggests there may be substantial undiscovered species diversity in this genus in other parts of Asia.

## Supplementary Material

XML Treatment for Metathrinca

XML Treatment for Metathrinca argentea

XML Treatment for Metathrinca bispina

XML Treatment for Metathrinca breviprocessa

XML Treatment for Metathrinca crassiprocessa

XML Treatment for Metathrinca digitiloba

XML Treatment for Metathrinca fopingensis

XML Treatment for Metathrinca fortispina

XML Treatment for Metathrinca graciliphalla

XML Treatment for Metathrinca intacta

XML Treatment for Metathrinca licina

XML Treatment for Metathrinca lingugnathos

XML Treatment for Metathrinca meihuashana

XML Treatment for Metathrinca mutativalvis

XML Treatment for Metathrinca nigrivenosa

XML Treatment for Metathrinca punctuatella

XML Treatment for Metathrinca sublunata

XML Treatment for Metathrinca tsugensis

XML Treatment for Metathrinca zhengi

## Additional information

### Artificial Intelligence (AI) use

The authors accept full responsibility for the content of the manuscript, including the disclosure of any use of AI.

No AI tools were used in the preparation of this manuscript.

### Author ORCIDs

S. Tang https://orcid.org/0009-0000-5575-1504

H. Li https://orcid.org/0000-0002-8953-3422

### Data availability

All of the data that support the findings of this study are available in the main text.
